# The spatiotemporal trend of human brucellosis in China and driving factors using interpretability analysis

**DOI:** 10.1038/s41598-024-55034-4

**Published:** 2024-02-28

**Authors:** Xiaohui Wen, Yun Wang, Zhongjun Shao

**Affiliations:** 1 Department of Epidemiology, Air Force Medical University, Xi’an, 710000 China; 2 Central Sterile Services Department, The First Affiliated Hospital of Xi’an Jiaotong University, Xi’an, 710000 China

**Keywords:** Public health, Risk factors, Environmental impact, Environmental impact

## Abstract

Human brucellosis has reemerged in China, with a distinct change in its geographical distribution. The incidence of human brucellosis has significantly risen in inland regions of China. To gain insights into epidemic characteristics and identify factors influencing the geographic spread of human brucellosis, our study utilized the Extreme Gradient Boosting (XGBoost) algorithm and interpretable machine learning techniques. The results showed a consistent upward trend in the incidence of human brucellosis, with a significant increase of 8.20% from 2004 to 2021 (95% CI: 1.70, 15.10). The northern region continued to face a serious human situation, with a gradual upward trend. Meanwhile, the western and southern regions have experienced a gradual spread of human brucellosis, encompassing all regions of China over the past decade. Further analysis using Shapley Additive Explanations (SHAP) demonstrated that higher Gross Domestic Product (GDP) per capita and increased funding for education have the potential to reduce the spread. Conversely, the expansion of human brucellosis showed a positive correlation with bed availability per 1000 individuals, humidity, railway mileage, and GDP. These findings strongly suggest that socioeconomic factors play a more significant role in the spread of human brucellosis than other factors.

Brucellosis, caused by the bacterium Brucella, is a zoonotic disease that affects both humans and animals worldwide, except for a few industrialized countries^1^. Various animal species have been identified as primary hosts of Brucella, including domestic animals such as goats, sheep, and pigs^2,3^. Brucellosis in animals can lead to miscarriage, infertility, and decreased milk production. Moreover, the high frequency of abortions in infected animals caused by brucellosis significantly impacts livestock production^4^. The disease is transmitted to humans through direct contact with infected animals or their excretions^5^. The common symptoms of human brucellosis include fever, chills, night sweats, fatigue, weakness, joint pain, muscle aches, and gastrointestinal problems^6^. The most effective way to prevent brucellosis is to avoid contact with infected animals, whether through livestock management or encounters with wildlife. Researchers have discovered that more than 500,000 new human cases are reported annually^7^. Thus, brucellosis has imposed a substantial burden on both public health and economies worldwide. Governments and health agencies across the globe have made concerted efforts to control and combat the spread of this disease^8–10^. However, brucellosis remains a neglected zoonotic disease, causing substantial harm to the health and economies of affected countries, and continues to be a serious global public health problem^11^. In recent years, there has been a resurgence of human brucellosis in China, characterized by a noticeable spatiotemporal trend. Human brucellosis remains a major public health concern, with outbreaks reported in 25 out of China’s 32 provinces or autonomous regions^12–14^. The incidence of human brucellosis was particularly high in northern China. However, there has been a notable shift in the geographic distribution of the disease, with an expansion toward urban areas in the southern inland regions of the country^15^. This change in distribution highlights the need for increased surveillance and control measures to effectively address the spread of human brucellosis in these newly affected areas.

China has a wide variety of ecological, environmental, meteorological, and economic landscapes throughout the country. The epidemiological characteristics and causes of human brucellosis vary, especially as the area has significantly different landscapes^16–22^. Previous studies have focused on the incidence of human brucellosis in mainland China, which has been instructive and illuminating for the prevention and control of human brucellosis^20,23,24^. However, very few investigations have investigated the spatiotemporal expansion characteristics and identified the driving factors of human brucellosis in China. In this study, we combined meteorological factors, socioeconomic factors, and agricultural and livestock factors to explore the characteristics of spatiotemporal expansion at the provincial level. Then, the Shapley Additive Explanation (SHAP) was used to explain the driving factors. These findings may provide more comprehensive insight into the reasons behind the spatiotemporal expansion of human brucellosis, as opposed to the declining trend observed in most infectious diseases. Therefore, these findings can potentially contribute valuable ideas and strategies to effectively curb the spread of human brucellosis.

## Results

### The spatiotemporal trend of human brucellosis in mainland China between 2004 and 2021

**Figure 1:**
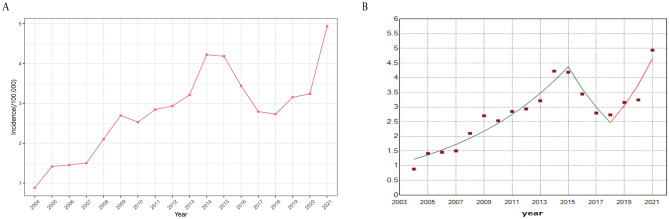
The incidence of human brucellosis exhibited a general upward trend from 2004 to 2021. (**A**) Line chart of human brucellosis incidence in mainland China from 2004 to 2021. (**B**) Joint point plot of human brucellosis incidence. The temporal change in incidence showed a trend of first increasing, decreasing, and then increasing.

Between 2004 and 2021, China reported a total of 684,293 cases of human brucellosis, resulting in an average annual reported incidence rate of 2.8 cases per 100,000 individuals. Over the years, the incidence of human brucellosis has consistently shown an upward trend (Fig. 1A). The join point regression model analysis revealed that the percentage of human brucellosis cases increased by an average of 8.2% (95% CI: 1.70%, 15.1%) from 2004 to 2021. Further phased analysis revealed that the incidence of human brucellosis increased by 12.3% annually (95% CI: 9.1%, 15.7%) from 2004 to 2015. Subsequently, from 2015 to 2018, there was a gradual decline in the incidence, with an annual percentage decrease of −17.3% (95% CI: −41.8%, 17.5%). However, from 2018 to 2021, there was another upward trend in the incidence in human brucellosis, with an annual percentage increase of 23.5% (95% CI: 5.8%, 44.2%) (Fig. 1B).

**Figure 2:**
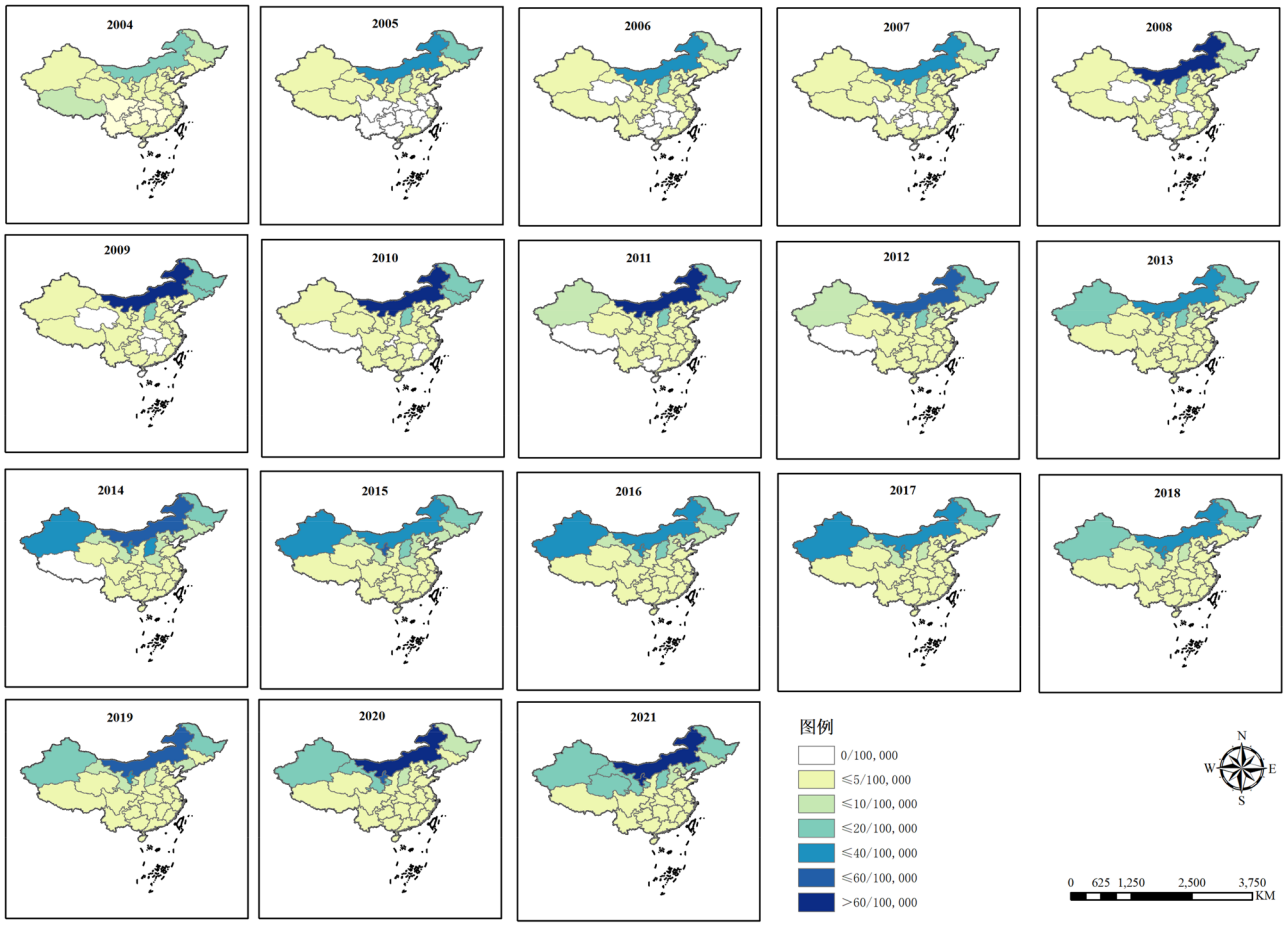
Geographic distribution of the annual incidence per 100,000 residents of human brucellosis from 2004 to 2021 in mainland China.

**Figure 3:**
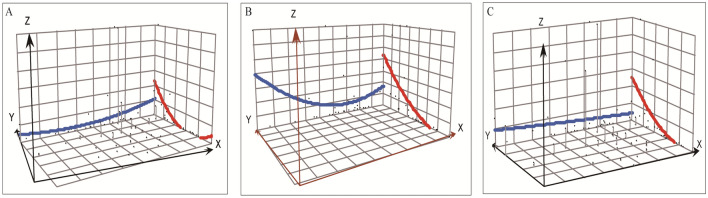
The spatial distribution trend of reported human brucellosis incidence in mainland China over different time periods. (**A**) From 2004 to 2015, an arch-shaped pattern was observed, with a higher incidence in the northern region and a decreasing trend from east to west. (**B**) From 2015 to 2018, the incidence of human brucellosis exhibited a decreasing trend from north to south, accompanied by an arch-shaped pattern from east to west. The western region had a higher incidence, suggesting a higher burden of the disease in western areas. (**C**) From 2018 to 2021, there was a continued decrease in the incidence of human brucellosis from north to south, as well as a gradual decreasing trend from west to east.

In terms of spatial distribution, the annual reported incidence of human brucellosis varied significantly across the 31 provinces, ranging from 0 to 88.61 cases per 100,000 people (Fig. 2). As shown in Fig. 2, Inner Mongolia Autonomous Region has the highest incidence rate of human brucellosis, followed by Shanxi Province, Heilongjiang Province, and Xinjiang Uygur Autonomous Region. From 2004 to 2015, an arch-shaped pattern was observed, with a higher incidence in the northern region and a decreasing trend from east to west. This indicated a concentration of cases in the northern provinces and a gradual decrease from east to western regions (Fig. 3A). Similarly, from 2015 to 2018, there was a decline in the incidence of human brucellosis from north to south, while an arch trend was observed from east to west, with the western region experiencing a higher incidence (Fig. 3B). Furthermore, from 2018 to 2021, there was a continued decrease in the incidence of human brucellosis from north to south, accompanied by a decreasing trend from west to east (Fig. 3C). These findings shed light on the spatial disparities in the incidence of human brucellosis across different regions of China. It is evident that the northern region has been severely impacted by human brucellosis, and this trend persists. However, there has been a gradual expansion of human brucellosis in the southern and western regions, progressively spreading to all corners of China. It is important to note that the northern regions continue to face a severe epidemic, with a high number of reported cases. In contrast, the incidence of human brucellosis has steadily increased in the western and southern regions, indicating a concerning trend of its spread.

### Prediction models for spatiotemporal expansion of human brucellosis

**Figure 4:**
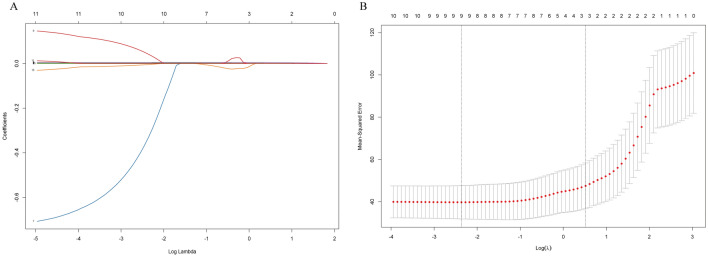
Feature selection was performed using the LASSO model. (**A**) A coefficient profile plot was generated by plotting the coefficients against the log (λ) sequence. The optimal λ value was determined, which resulted in 11 nonzero coefficients. A vertical line was drawn at this selected value to indicate its significance. (**B**) The deviance was generated against log (λ). The minimal criteria and the 1 standard error (1-SE) criteria were employed to identify the optimal values. Dotted vertical lines were drawn at these optimal values. In this analysis, a log (λ) value of 0.005 was chosen, which corresponded to a minimal criteria value of 0.059.

Using the lasso method in the selection of predictors, we identified a total of 11 predictors that were found to have a significant impact on predicting the spread of human brucellosis (Fig. 4). These predictors encompassed a range of variables, including the number of hospital beds per 1000 individuals, railway mileage, Gross Domestic Product (GDP), GDP per capita, humidity, number of sheep, education funds, road mileage, temperature, goat population, and healthcare personnel.

To develop our prediction models, we utilized the Extreme Gradient Boosting (XGBoost) algorithm, a widely recognized statistical model known for its accuracy and reliability. By harnessing the power of the XGBoost algorithm, our objective was to effectively predict and comprehend the spatial patterns of human brucellosis. We divided our dataset into two parts, with 80% of the data allocated for training and the remaining 20% for testing. The testing dataset yielded results indicating that the XGBoost model attained an accuracy of 0.89 (95% CI: 0.81, 0.95). The model also showed a sensitivity of 0.94 and a specificity of 0.55, while the AUC was calculated to be 0.85.

### Interpretability analysis

**Figure 5:**
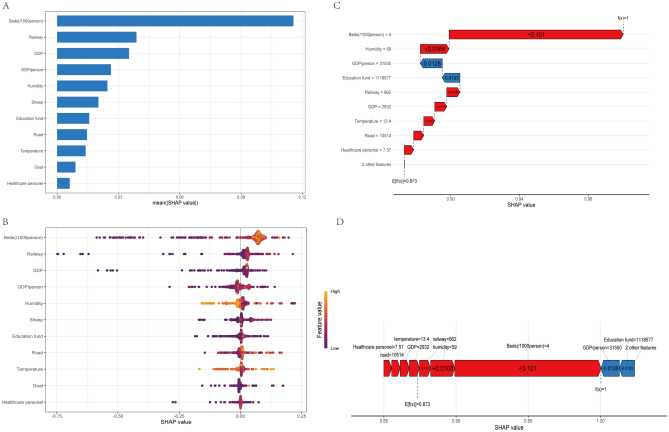
Importance of predictors for human brucellosis expansion using SHAP value values in the best model with different interpretability plots. (**A**) The bar chart shows the SHAP values for each driver in descending order of their mean importance values. The height of each bar indicates the magnitude of the SHAP value. (**B**) The SHAP summary plot provides a visual representation of the range and density of the SHAP values, which depict the distribution of each feature’s effect on the best model outputs. Each dot on the plot represents a case in the dataset, with the color of the dot indicating the feature’s value, ranging from purple (lowest) to yellow (highest). The horizontal axis displays the corresponding SHAP value of the feature, with positive values contributing to the prediction of occurrence and negative values predicting the opposite. The purple to yellow dots represents low to high values of each predictor. The x-axis shows the SHAP value, indicating the contribution of each predictor to the predicted probability of human brucellosis, with positive values predicting a higher probability and negative values predicting a lower probability. (**C**) Waterfall plot illustrating the cumulative effect of each driver on the predicted outcome. It showed how each driver contributes to the overall prediction by stacking the SHAP values in a cascading manner. Positive values indicate drivers that increase the predicted outcome, while negative values indicate drivers that decrease it. (**D**) Force plot presents the individual contributions of each driver to the predicted occurrence of human brucellosis. It visualizes the direction and magnitude of the SHAP values for each driver. The color scheme of the chart represents the value of each feature, with red indicating a positive correlation with the occurrence of human brucellosis, blue indicating a negative correlation, and the length of each column representing the weight size of the feature’s influence.

The SHAP values of the best model are summarized in Fig. 5A,B to highlight the importance of its predictors. Analyzing the mean SHAP value, it was determined that the top four predictors of human brucellosis, in order of significance, were the number of hospital beds per thousand individuals, railway mileage, GDP, and GDP per capita. This finding suggests that socioeconomic factors have a greater impact on the spread of human brucellosis than other factors.

The force plot and waterfall plot provided further insights into the expansion of human brucellosis. Surprisingly, higher GDP per capita and increased education funding were found to decrease the spread of human brucellosis. On the other hand, the expansion of human brucellosis showed a positive correlation with the availability of hospital beds per 1000 individuals, humidity levels, railway mileage, GDP, and other predictors, as depicted in Fig. 5C,D. These results clearly indicate that regarding the spatial expansion of human brucellosis, socioeconomic factors play a more significant role than livestock factors.

**Figure 6:**
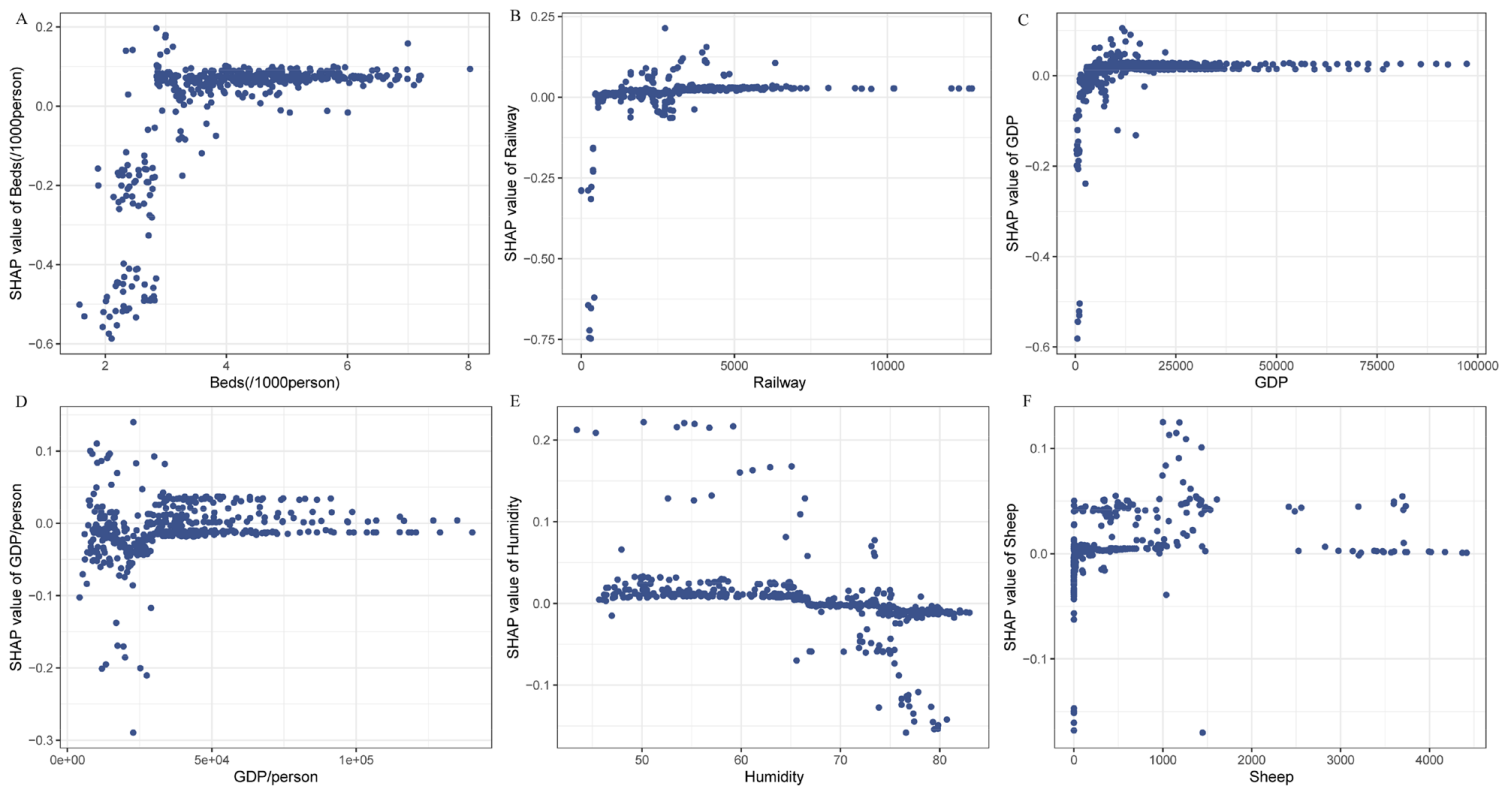
SHAP decency plot between human brucellosis expansion and individual risk factors for the six most important predictors in order. (**A**) Dependence plot between the number of beds per 1000 people and SHAP value. (**B**) Dependence plot between railway mileage and SHAP value. (**C**) Dependence plot between GDP and SHAP value. (**D**) Dependence plot between GDP per capita and SHAP value. (**E**) Dependence plot between humidity and SHAP value. (**F**) Dependence plot between sheep number and SHAP value.

In addition to analyzing the predictors, we also generated SHAP dependence plots for human brucellosis expansion and the 6 most important variables. The SHAP values gradually decreased with increasing GDP per capita, implying that low GDP per capita values had a positive effect on predicting human brucellosis occurrence (Fig. 6D). In contrast, increasing values of the number of hospital beds per 1,000 people, railway mileage, GDP, humidity, and sheep number showed a positive correlation with increasing SHAP values (Fig. 6A–C,E,F). These findings further emphasize the significance of socioeconomic factors in predicting the spread of human brucellosis.

## Discussion

From 2004 to 2021, the reported incidence of human brucellosis exhibited significant variation, with an average annual incidence of 2.81 cases per 100,000 people. The incidence ranged from 0 to 88.62 cases per 100,000 residents during this period. A detailed analysis indicated that, on average, the percentage of human brucellosis cases increased by 8.2% from 2004 to 2021. Further examination of the data revealed an upward trend in the incidence of human brucellosis from 2004 to 2015, followed by a gradual decline from 2016 to 2018 and another increase from 2019 to 2021. In terms of spatial distribution, the annual reported incidence varied greatly among the 31 provinces, with rates ranging from 0 to 88.62 cases per 100,000 individuals. The northern regions consistently exhibited a high incidence of human brucellosis, indicating a severe epidemic in those areas. Meanwhile, there has been a steady increase in the incidence of human brucellosis in the western and southern regions, suggesting a concerning trend of the disease spreading to all areas. These spatiotemporal trends highlight the varying patterns of human brucellosis incidence across different regions of China over the years, providing valuable insights for targeted prevention and control strategies in specific areas. Research has shown that the incidence of human brucellosis is closely related to livestock density, especially sheep and goats^14^. Higher incidences of human brucellosis tend to occur most commonly in grasslands at moderate elevations, where sheep and goats are the predominant livestock^25,26^. In China, the northern region significantly contributes to the country’s goat and sheep population, thereby further accelerating the spread of the disease. Notwithstanding the fact that the Northwest and Northeast regions of China represent the country’s primary goat and sheep production bases, human brucellosis incidences display certain variances due to variations in climate, natural environment, and economic development level^17,27^.

To investigate the relationship between various contributing factors and the spread of human brucellosis in different geographic regions, we employed the XGBoost machine learning method in our research. The XGBoost classification technique proved highly effective in identifying significant patterns and developing case-based reasoning algorithms, which have been widely utilized in statistical analysis^28^. To gain insights into the inner workings of the machine learning model, we utilized the SHAP framework, which serves as a valuable tool for unraveling the “black box” nature of machine learning. This framework enabled us to explain how the model operates by examining the impact of individual features^29,30^. Through this analysis, we successfully identified several influential factors that have the potential to explain the expansion of human brucellosis. These findings provide valuable insights for further understanding and addressing the spread of this disease. Our SHAP analysis revealed that socioeconomic drivers may play a vital role in the expansion of human brucellosis, which is consistent with previous findings^17,24^. Attention should be focused on socioeconomic factors, particularly beds per thousand individuals, rail miles, GDP, and GDP per capita^31–33^.

Previous studies have indicated that countries with lower GDP tend to experience more severe cases of human brucellosis^20,34^. This can be attributed to the fact that high-income countries have better resources and financial support to implement effective disease prevention and control measures. Higher GDP also contribute to improved sanitary conditions and increased access to medical treatment, which in turn increases the likelihood of detecting diseases. Increased GDP can lead to higher investment in infrastructure, which is instrumental in improving the living standards of residents and enhancing the accessibility of medical resources. On the other hand, as the standard of living in China has improved, there has been an increased demand for animal protein, leading to a rise in livestock production, slaughter, and transportation for meat consumption^22^. Consequently, more individuals contact animal hosts and their vectors, potentially increasing the risk of disease transmission to humans^35^. In our study, we used the number of hospital beds as a proxy variable to measure the medical level of an area. This is consistent with previous findings that areas with low levels of medical services and limited medical resources may have a higher occurrence of zoonotic diseases^18,20,36^.

The impact of the public transportation system, specifically railway and road mileage, has been extensively discussed in relation to the spread of diseases such as COVID-19 and influenza^37–39^. However, the influence of public transportation on the expansion of human brucellosis has not been thoroughly explored. This presents an intriguing avenue for future research to investigate how public transportation may contribute to the spread of human brucellosis.

Furthermore, the increase in residents’ education funds has led to improvements in education levels and heightened disease awareness. As individuals become more educated, they are more likely to be aware of diseases and take necessary precautions^22^. Increased investment in education can enable a wider range of individuals to acquire an education, leading to a higher level of health and disease awareness among the population. This, in turn, can significantly reduce the transmission and spread of human brucellosis.

Our study found that human brucellosis expansion can potentially be mitigated by higher GDP per capita, as increased economic prosperity has been shown to improve education levels and raise awareness of the disease. This, in turn, contributes to a reduction in the spread of brucellosis. Understanding the relationship between education, economic development, and disease awareness can provide valuable insights into public health strategies and interventions against human brucellosis. Several previous studies indicated that an increased number of sheep and cattle were responsible for an increased human brucellosis incidence^16,17,27^, which potentially drove the emergence and spatial expansion of human brucellosis in China, and our results supported this conclusion. However, our study demonstrates that livestock factors were less important for the spatiotemporal expansion of human brucellosis than socioeconomic factors.

This study investigated the spatiotemporal trend of human brucellosis and explored the driving factors contributing to its spatiotemporal spread using the SHAP framework. However, it is essential to acknowledge the limitations of our research. First, the use of passive surveillance data may not provide a complete picture of the disease’s incidence compared to active surveillance methods. Passive surveillance data rely on the voluntary reporting of cases by healthcare providers, which could result in underreporting, especially for cases with mild clinical symptoms. Consequently, some cases may have been missed, leading to an underestimation of the true incidence of the disease. The study’s data was collected from the National Infectious Disease Reporting System, a passive surveillance system that relies on healthcare providers to voluntarily report cases. Although the system accommodates a broad spectrum of disease reporting, voluntary reporting may lead to underreporting. This is especially evident in milder cases of human brucellosis, where symptoms can be nonspecific and might not be promptly detected. Consequently, the reported cases may potentially underestimate the actual occurrence of the disease. Second, the research data used in this study were gathered from provincial administrative regions and presented as the provincial average. As a vast country, China encounters diverse challenges that differ significantly across its numerous county and municipal areas within the same province. Factors such as economic development, population size and movement, and health conditions could all influence the incidence of human brucellosis. It is important to recognize that these factors may vary within provinces, potentially influencing the incidence of human brucellosis. Despite these limitations, our study provides valuable insights into the expansion of human brucellosis and highlights the importance of considering socioeconomic factors. Our findings could be used to inform public health policies and interventions to reduce the burden in affected communities.

## Conclusions

Our study showed a consistent upward trend in the incidence of human brucellosis, with a significant increase of 8.20% from 2004 to 2021 (95% CI: 1.70, 15.10). Moreover, the northern region continues to face a serious human brucellosis situation, showing a gradual upward trend. Meanwhile, the western and southern regions have experienced a gradual spread of human brucellosis, encompassing all regions of China in the past decade. Our research demonstrated that human brucellosis has experienced a resurgence in China, accompanied by a notable shift in its geographic distribution. Initially, the disease was predominantly concentrated in the traditional livestock regions of the country. Then, there was a significant expansion of human brucellosis cases toward the inland areas of China. Further analysis using SHAP demonstrated that higher GDP per capita and increased funding for education have the potential to reduce the spread of human brucellosis. Conversely, the expansion of human brucellosis showed a positive correlation with bed availability per 1000 individuals, humidity levels, railway mileage, and GDP. These findings strongly indicate that socioeconomic factors play a more significant role in the spread of human brucellosis than other factors. Overall, these findings underscore the significance of taking socioeconomic factors into account when studying and tackling the spread of human brucellosis in China. For example, we should increase education funding for the popularization of basic knowledge of infectious diseases, improve the accessibility of the infrastructure, and allow more people to enjoy better medical resources.

## Materials and methods

### Human brucellosis

The China Public Health Science Data Center obtained the data of human brucellosis in China mainland from 2004 to 2019, including the number of cases, the incidence rate, and the provinces where the cases occurred. The human brucellosis incidence from 2020 to 2021 was obtained from the Yearbook of Health Statistics. The number of cases of human brucellosis is calculated based on the year-end resident population data.

To provide geographical context, we downloaded an electronic map of China at a scale of 1:1,000,000 from the National Earth System Science Data Sharing Platform. It is worth noting that human brucellosis is classified as a notifiable class B infectious disease in China, and as such, all diagnosed cases must be reported to the China National Notifiable Infectious Disease Surveillance System (NNDSS) within 24 h. The diagnosis of human brucellosis in China adheres to the guidelines outlined in the Law of Communicable Diseases Prevention and Control, a comprehensive guidebook published by the National Health Commission of the People’s Republic of China. The diagnosis involves considering both the individual’s epidemiological history and clinical manifestations to ensure a thorough and accurate assessment of the condition^40^.

### Driving factors

The meteorological data, including monthly precipitation (mm), maximum wind speed (m/s), minimum temperature (°C), and humidity (%), were obtained from monitoring stations of the China Meteorological Data Service Center (CMDC). These data were collected for each province in mainland China from 2004 to 2021. Elevation data with a resolution of 90 m were extracted from the Shuttle Radar Topography Mission (SRTM). Landcover data obtained from the European Space Agency (ESA) WorldCover dataset were available at a resolution of 0.3 s. All socioeconomic indicators were extracted from the China Statistical Yearbook, which is published by the Chinese National Bureaus of Statistics.

### Temporal trend analysis

In this study, joint point regression was employed to detect and describe trend analysis. This statistical method has been shown to be effective in helping researchers analyze changes in time trends and identify significant turning points in the data^41^. Joint point regression offers researchers an intuitive approach to analyze changes in time trends and gain insights into significant turning points within the data. This method utilizes segmented linear regression models to fit the data, dividing the time series into distinct linear segments. Each segment is characterized by its own slope and intercept. By comparing the slopes and intercepts across different segments, joint point regression can effectively identify change points in the data and provide estimates for the timing and magnitude of each change point^42^. The variation in temporal trends was described by analyzing the annual percent change (APC), the average annual percent change (AAPC), and the 95% confidence interval using a join point regression model. The joint point regression analysis in this study was conducted using the Joint Point Regression Program (version 5.0.2), which was developed by the National Cancer Institute in Bethesda, MD, USA.

### The spatial trend analysis

Spatial trend analysis is a technique used to analyze patterns and trends in spatial data by visualizing attribute values of interest on a 3D map. This is achieved by elevating the points on the map to a height corresponding to the attribute value, with the points projected onto a plane in two perpendicular directions (north and west). Spatial trend analysis is an advanced technique that employs polynomial interpolation to create a smooth surface based on a set of sample points. The process involves the application of polynomial regression to create a raster that optimally fits a least square surface, with the degree of the polynomial being adjustable to tailor the surface to specific requirements^43,44^. In this study, The X and Y axis represent the geometric center of specific study region, and Z-axis represents the incidence of human brucellosis^16^. The geographic distribution map and spatial trend analysis were performed using ArcGIS 10.2.

### Feature selection and hyperparameter optimization

Feature selection in our study was conducted through a combination of literature review and algorithmic approaches. We first conducted a comprehensive review of relevant studies to identify influential factors mentioned in previous papers (Supplemental Table 1). To further refine the feature selection process, we utilized the least absolute shrinkage and selection operator (LASSO) technique. This method shrinks the coefficients of irrelevant variables to zero by applying a penalty term to the sum of their absolute values^45^. By doing so, it encourages the model to select only the most important variables. tenfold cross validation is used in LASSO regression variable screening to ensure the stability of the model. The Lasso model identifies relevant features by detecting nonzero coefficients and determining the optimal λ value (λ = 0.059) based on minimal criteria^46^. The glmnet package is used for lasso regression variable filtering.

After feature selection, we employed the XGBoost machine learning method, which is widely recognized and highly regarded in the fields of data mining and statistics. This algorithm has gained popularity and has been voted as one of the top ten data mining algorithms due to its straightforward implementation and excellent classification performance^47^. The dataset was randomly divided into training and testing sets at a ratio of 8:2. The training set was used for model development, while the test set was used for model validation and evaluation. The tenfold cross-validation (CV) technique was applied to search for optimal hyperparameters through a random search. The selection of optimal hyperparameters was based on maximizing the accuracy of each model. To assess the performance of the model, various metrics, such as accuracy, sensitivity, specificity, and the area under the receiver operating characteristic curve (AUC), were calculated. These metrics provide insights into the model’s accuracy, its ability to correctly identify positive and negative cases, and its overall discriminatory power^48^. The overall predictive accuracies of the developed prediction models were evaluated using the AUC of the ROC curve, where a higher AUC indicates better prediction performance.

### Interpretability analysis with SHAP

The SHAP framework has emerged as a recent development for evaluating predictions generated by complex black box machine learning algorithms. It is based on the theoretically optimal Shapley values of the game^49^. Research has shown that SHAP values outperform other local explanation methods, including computationally expensive model-agnostic alternatives, when interpreting large datasets and decision trees^50^. In this study, we developed optimal models to investigate the influential predictors in predicting the expansion of human brucellosis. We employed SHAP values to explain the predicted values by quantifying the contribution of each predictor. By analyzing the SHAP values, we can examine the relationship between variables and the occurrence of human brucellosis. A positive SHAP value > 0 suggests that the variable positively contributes to the predicted value and influences the outcome. Conversely, a negative SHAP value < 0 indicates that the variable negatively affects the predicted value, exerting an inhibitory effect on the outcome. To comprehensively analyze the impact of different drivers on the spread of human brucellosis, we utilized various forms of SHAP values, including bar plots, beeswarm plots, waterfall plots, force plots, and dependency plots. By leveraging these diverse forms of SHAP values, we can acquire a comprehensive and in-depth understanding of how different drivers influence the spread of human brucellosis. The XGboost package is used to train the model, and the shapviz package visualizes shap values.

All statistical analyses and the development of prediction models were performed using R software (version 4.2.2).

### Supplementary Information

Supplementary Information.

## Data Availability

The dataset analyzed during the current study was collected from a public database in China and is publicly available online (China Public Health Science Data Center: https://www.phsciencedata.cn/Share/, the Yearbook of Health Statistics: http://www.nhc.gov.cn/mohwsbwstjxxzx/tjzxtjcbw/tjsj_list).
