# Supplementary material for: The spatiotemporal trend of human brucellosis in China and driving factors using interpretability analysis
Source: Sci Rep. 2024 Feb 28;14:4880. doi: 10.1038/s41598-024-55034-4 (PMC10901783; doi:10.1038/s41598-024-55034-4)
Supplement: Supplementary file 1 — Supplementary Information. [file 41598_2024_55034_MOESM1_ESM.pdf]

Supplement table 1. The previous papers reported influencing factors of human brucellosis.

Agricultural and livestock drivers	Geographic and meteorological drivers	Socio-economic drivers	References
sheep, goats, swine,	Elevation, Grassland	-	Epidemiological features and risk factors associated with the spatial and temporal distribution of human brucellosis in China[1].
sheep, mutton_ production,	-	GDP, highway, number of medical institutions, public health expenditure, and rural medical expenditure proportion	An exploratory study of factors associated with human brucellosis in mainland China based on time-series-cross-section data from 2005 to 2016[2]
Sheep, cattle	Rainfall, precipitation, temperature	GDP, population density	Spatial-temporal distribution of human brucellosis in mainland China from 2004 to 2017 and an analysis of social and environmental factors[3]
sheep, cattle	temperature, wind speed	GDP, hospital beds	Spatiotemporal distribution of human brucellosis in Inner Mongolia, China, in 2010–2015, and influencing factors[4]
Sheep, cattle	-	-	Spatio-temporal cluster and distribution of human brucellosis in Shanxi Province of China between 2011 and 2016[5]
-	Temperature, frosty days	-	A primary investigation of the relation between the incidence of brucellosis and climatic factors in Iran[6]
Sheep, cattle	Precipitation, Mean maximum temperature, Elevation, NDVI, precipitation, humidity, evaporation	population	Spatial prediction of human brucellosis (HB) using a GIS-based adaptive neuro-fuzzy inference system (ANFIS)[7]
-	Temperature, precipitation, NDVI,	-	Driving effect of multiplex factors on human brucellosis in high incidence region, implication for brucellosis based on one health concept[8]
-	Temperature, sunshine duration, atmosphere pressure	-	Influence and prediction of meteorological factors on brucellosis in a northwest region of China[9]
Sheep	SO_2, temperature,	consumption of meat per capita	Spatiotemporal Pattern Evolution and Driving Factors of Brucellosis in China, 2003–2019[10]
number of farms	-	population density, GDP, roads, nighttime lights	Epidemic characteristics and transmission risk prediction of

			brucellosis in Xi'an city, Northwest China[11]
-	Temperature, sunshine duration, rainfall, relative humidity, and evaporation	-	Spatiotemporal expansion of human brucellosis in Shaanxi Province, Northwestern China, and model for risk prediction[12]

References

1 Li, Y. J., Li, X. L., Liang, S., Fang, L. Q. & Cao, W. C. Epidemiological features and risk factors associated with the spatial and temporal distribution of human brucellosis in China. *BMC Infect Dis* **13**, 547, doi:10.1186/1471-2334-13-547 (2013).

2 Lin, Y., Xu, M., Zhang, X. & Zhang, T. An exploratory study of factors associated with human brucellosis in mainland China based on time-series-cross-section data from 2005 to 2016. *PLoS One* **14**, e0208292, doi:10.1371/journal.pone.0208292 (2019).

3 Peng, C., Li, Y. J., Huang, D. S. & Guan, P. Spatial-temporal distribution of human brucellosis in mainland China from 2004 to 2017 and an analysis of social and environmental factors. *Environ Health Prev Med* **25**, 1, doi:10.1186/s12199-019-0839-z (2020).

4 Liang, D. *et al.* Spatiotemporal distribution of human brucellosis in Inner Mongolia, China, in 2010-2015, and influencing factors. *Sci Rep* **11**, 24213, doi:10.1038/s41598-021-03723-9 (2021).

5 Wang, T. *et al.* Spatio-temporal cluster and distribution of human brucellosis in Shanxi Province of China between 2011 and 2016. *Sci Rep* **8**, 16977, doi:10.1038/s41598-018-34975-7 (2018).

6 Dadar, M., Shahali, Y. & Fakhri, Y. A primary investigation of the relation between the incidence of brucellosis and climatic factors in Iran. *Microb Pathog* **139**, 103858, doi:10.1016/j.micpath.2019.103858 (2020).

7 Babaie, E., Alesheikh, A. A. & Tabasi, M. Spatial prediction of human brucellosis (HB) using a GIS-based adaptive neuro-fuzzy inference system (ANFIS). *Acta Trop* **220**, 105951, doi:10.1016/j.actatropica.2021.105951 (2021).

8 Peng, R. *et al.* Driving effect of multiplex factors on human brucellosis in high incidence region, implication for brucellosis based on one health concept. *One Health* **15**, 100449, doi:10.1016/j.onehlt.2022.100449 (2022).

9 Zheng, H. *et al.* Influence and prediction of meteorological factors on brucellosis in a northwest region of China. *Environmental Science and Pollution Research* **30**, 9962-9973, doi:10.1007/s11356-022-22831-1 (2023).

10 Xu, L. & Deng, Y. Spatiotemporal Pattern Evolution and Driving Factors of Brucellosis in China, 2003-2019. *Int J Environ Res Public Health* **19**, doi:10.3390/ijerph191610082 (2022).

11 Zhao, C. *et al.* Epidemic characteristics and transmission risk prediction of brucellosis in Xi'an city, Northwest China. *Front Public Health* **10**, 926812, doi:10.3389/fpubh.2022.926812 (2022).

12 Yang, Z. *et al.* Spatiotemporal expansion of human brucellosis in Shaanxi Province, Northwestern China and model for risk prediction. *PeerJ* **8**, e10113, doi:10.7717/peerj.10113

(2020).
